# Social and Preventive Factors That Explain Oral Health among Pregnant Women in the Canton of Cuenca, Ecuador

**DOI:** 10.3390/healthcare11192664

**Published:** 2023-10-01

**Authors:** Milton Fabricio Lafebre-Carrasco, Millán Arroyo-Menéndez, David Lozano-Paniagua, Tesifón Parrón-Carreño, Bruno José Nievas-Soriano

**Affiliations:** 1 School of Dentistry, Cuenca University, Cuenca 010105, Ecuador; fabricio.lafebre@ucuenca.edu.ec; 2 Department of Sociology: Theory and Methodology, Faculty of Political Science and Sociology, Member of TRANSOC Research Institute, Complutense University of Madrid, 28223 Madrid, Spain; millan@cps.ucm.es; 3 Department of Nursing, Physiotherapy and Medicine, University of Almeria, 04120 Almeria, Spain; tpc468@ual.es (T.P.-C.); brunonievas@ual.es (B.J.N.-S.)

**Keywords:** oral health, pregnant women, children, dentist, disadvantaged regions, dental diseases

## Abstract

(1) Background: pregnant women in underprivileged areas may face challenges that affect their oral health. The analysis of these issues such as toothaches or cavities, among others could be crucial for them. However, no studies have been conducted in Cuenca, Ecuador. Thus, this study aimed to create a model explaining how social factors and healthy habits impact oral health in Cuenca, Ecuador. (2) Methods: An observational study was performed using a questionnaire developed from scratch. Principal component factor analysis was performed to calculate the oral disease index based on the oral health issues reported by women during pregnancy. (3) Results: 1971 women participated in the research. In total, 88% reported at least one oral health problem, with cavities (34%) and bleeding gums (33%) as the most prevalent. The rate of preventive visits and frequent brushing were the two variables that most impacted the oral disease index. The consumption of sweets, age, and the belief that visiting the dentist harms their unborn child were also important factors. However, income, education, and ethnic background showed little to no effect. (4) Conclusions: The most beneficial determinants of oral health factors in pregnant women in Cuenca, Ecuador, are preventive dentist visits, frequent brushing, and a contained consumption of sweets. The main harmful factors are age and the misconception that dental visits can harm their unborn child. Surprisingly, income, education, and ethnic background have little effect. This study can be replicated in other countries and cultures.

## 1. Introduction

Pregnancy is usually associated with oral health problems [1,2,3,4]. During this process, hormonal changes can cause local alterations, affecting the pH levels and oral microbiota, leading to a higher frequency of oral pathologies [4,5]. Some authors have highlighted that certain oral conditions during pregnancy can adversely affect the mother and developing fetus [6]. Numerous variables and factors are associated with oral health in the general population [7,8]. The older the age, the greater the tendency to present oral health problems [9,10]. The general population also consumes more sugar per day than recommended, increasing the prevalence of dental caries [11]. During pregnancy, consuming more sweets can be associated with hormonal changes, increased energy needs, and emotional fluctuations [11]. Its consumption is associated with pathologies such as gestational diabetes and macrosomia of the newborn [12]. People with lower incomes have less access to health care [13,14]. Consequently, women earning less tend to have poorer oral hygiene practices [15,16]. There is also a direct relationship between educational level and oral health [14,17].

Particular beliefs can have an impact on one’s health. For example, the idea that losing teeth during pregnancy or dental procedures can be detrimental to pregnancy is expected. Furthermore, pregnant women should indulge in their cravings, although many involve sugary foods [15,18]. Specific authors suggest a direct connection between these beliefs and poor oral health during pregnancy [19,20]. Other authors suggest that being Indigenous may be associated with poor oral health [18,21]. However, other studies have not found such an association [20,22]. Lower attendance at the dental office during pregnancy was also described [3,23,24]. One of the barriers to attendance is fear or anxiety about dental treatment [25]. Visiting the dentist generates moderate to severe anxiety, predicting poor oral health [26,27], especially in women [28]. The fear of pain decreases dental interventions and preventive consultations [7,16].

However, improving oral hygiene in pregnant women has an impact on their general health [29], on their oral health during pregnancy [30], and on that of their children [17]. For example, adequate prevention during pregnancy can even reduce the incidence of childhood caries [6]. Therefore, it is essential to promote preventive habits [3,31]. In this sense, dental care visits, especially preventive ones are effective. These appointments help prevent or lessen oral problems and serve as a reminder to prioritize oral health education [32]. The frequency of preventive visits varies greatly depending on the level of development and the per capita income. Wealthier countries visit more frequently, while low-income countries have very few [33].

To improve oral health among pregnant women, we must consider several factors. Addressing these factors comprehensively is crucial, especially in underprivileged areas, to ensure mothers-to-be and newborns receive the necessary care and support for optimal oral health. Despite the precarious state of oral health in pregnant women in Cuenca, Ecuador, no studies were conducted to investigate the factors that may contribute to this issue from the perspective of these women. This issue presents a significant opportunity for improvement. Therefore, this study aimed to create a model explaining how social factors and healthy habits impact oral health in Cuenca, Ecuador. We also sought to examine if some social aspects could also impact the oral health of these women. Habits such as oral hygiene, preventive visits, sugary food consumption, and various social, demographic, educational, socioeconomic, and psychological variables were evaluated. Additionally, we examined how ethnicity, anxiety about experiencing pain during appointments, and concerns about harm to the fetus from dental procedures can affect the situation.

## 2. Materials and Methods

### 2.1. Study Design

An observational study was performed using a questionnaire developed from scratch. The questionnaire was based on a review of the literature and the professional experience of the authors of this investigation. The questionnaire collected exploratory data on various factors, including age, self-identification as Indigenous, net monthly household income, level of education, the frequency of brushing and use of oral rinses, and the frequency of sweet consumption. Furthermore, the questionnaire investigated whether participants believed that going to the dentist could harm their fetus and the correlation between pain, fear, going to the dentist, and the number of preventive dental visits made. The N/P3 index was calculated from the last question. N represented the number of problems the participant visited the dentist during his lifetime, so they could only cite those she knew. P3 was the total number of visits to the dentist. Higher values of the ratio value indicate poorer oral health among the population.

A pre-test was conducted with 30 female participants to evaluate the questionnaire’s ease of completion. The questionnaire took approximately 12 min to complete. The participants found the questions easy and the survey completion time acceptable.

### 2.2. Participants and Data Collection

A study conducted in Cuenca (Ecuador) involved 1971 pregnant women in 2017. Participants who attended morning or afternoon consults at gynecological care centers were invited to participate if they met the following inclusion criteria: pregnant women who understood Spanish and agreed to complete the survey. The survey was conducted face to face, using a structured, closed-ended paper questionnaire. One hundred trained medical students acted as recruiters and interviewers, helping in the process. Participants were fully informed about the study objectives and consented to participate. They also knew they could withdraw from the study at any time. No personal information was collected that could be used to directly or indirectly identify women who participated in the survey.

### 2.3. Questionnaire Validation Studies

Exploratory and confirmatory factorial analyses were performed to evaluate the validity of the construct. The adequacy of exploratory factor analysis (EFA) was determined by analyzing the Bartlett test and the Kaiser–Meyer–Olkin (KMO) measure. We evaluated the construct validity of the qualitative questionnaire items via exploratory factor analysis. Factor loadings and Cronbach’s alpha were examined to determine if an item was redundant or did not measure the same underlying construct, including or excluding the item via an iterative process [34,35]. Confirmatory factor analysis (CFA) was performed using the maximum likelihood estimation method. This method evaluated the items determined in the exploratory factor analysis. SPSS version 28 (IBM Inc., Armonk, NY, USA) was used for statistical analysis. For the confirmatory factor analysis, we used the AMOS version 28 statistical software (IBM Inc., Armonk, NY, USA).

### 2.4. Determination of the Oral Disease Index

The oral disease index was calculated using a principal component factor analysis of oral health issues reported by women at various times, including throughout their lifetime, in the previous two years, and during pregnancy. This index was assessed using a statistical reliability exploratory test based on Cronbach’s alpha [36]. Beforehand, the variables that were linked were correlated. For the ones that were not metric or did not meet normality conditions, Pearson and Spearman’s correlations were acquired. Various factors related to oral health were analyzed, including age, ethnic background, income, education level, brushing and rinsing habits, sweet intake, the fear of dental visits during pregnancy, anxiety related to dental visits, and the frequency of preventive dental check-ups. After identifying the significant variables, a path analysis model was constructed using structural equations to explain their association with the dependent variable.

### 2.5. Ethical Aspects

The research followed the ethical guidelines outlined in the Declaration of Helsinki. The anonymity of all participants was safeguarded during the collection, storage, and analysis of the data. No personal information was collected, and confidentiality was strictly maintained. The informed consent of the participants was signed, who were informed of the objectives of this investigation. All procedures mentioned in this research received approval from two ethics committees, as it was a collaboration between universities in Ecuador and Spain: the Research and Ethics Committee of the Department of Nursing, Physiotherapy and Medicine of the University of Almeria (Spain) with the approval number EFM 278/23; and the Bioethics Committee of the University of Cuenca, Ecuador, with the code 2017 008EO.

## 3. Results

The study involved 1971 women aged 14 to 46 years, with an average age of 26.8 years and an average of 0.8 children (range 0–8). An amount of 1,416 participants (74.9%) had a primary or secondary education level, and 1566 women (79.5%) reported monthly net income under $1000 (Table 1).

Almost all participants (99%) reported experiencing oral health problems at some point. The most common problems were cavities (84%) and toothaches (74%), which were also the main reasons for visiting the dentist (Table 2). On average, individuals visited the dentist 2.6 times in their lifetime. During pregnancy, 88% of women reported having at least one oral health problem, with cavities (34%), bleeding gums (33%), and toothaches (21%) being the most prevalent. On average, each woman experienced 1.3 oral health problems during pregnancy and visited the dentist 1.7 times.

Of all survey respondents, 66% brushed their teeth three times daily, 26% brushed twice daily, and 9% brushed once or less. Among the female participants, 31% used oral rinses once or twice a day, and 9% used them three times a day. Regarding sweet consumption, 40% of women reported eating sweets daily: 19% ate them once a day, 11% ate them twice a day, and 10% ate them three or more times a day. The N/P3 index produced a variable ranging from 0.75 to 1.0, with a mean of 0.83 and a standard deviation of 0.173. The oral disease index had a Cronbach’s alpha of 0.721. The KMO test for the principal component solution scored 0.691 (Table 3).

Table 4 shows the 26 items that combined the ten oral diseases stated by the participants.

As shown in Table 5, Table 6, Table 7 and Table 8, the analysis showed positive values that indicated oral disease, while negative values indicated oral health. Positive values suggested more oral health problems than average, while negative values indicated the opposite. On average, there were 1.32 mentions of oral problems during pregnancy, 1.86 in the last two years, and 3.64 throughout life, associated with the zero or mean value of the index.

After analyzing the dependent variable, oral disease, and all other variables in the questionnaire, it was found that there was no significant correlation between educational level and being Indigenous. However, there were low but statistically significant correlations between the dependent variable and the rest of the variables listed in Table 9.

**Figure 1:**
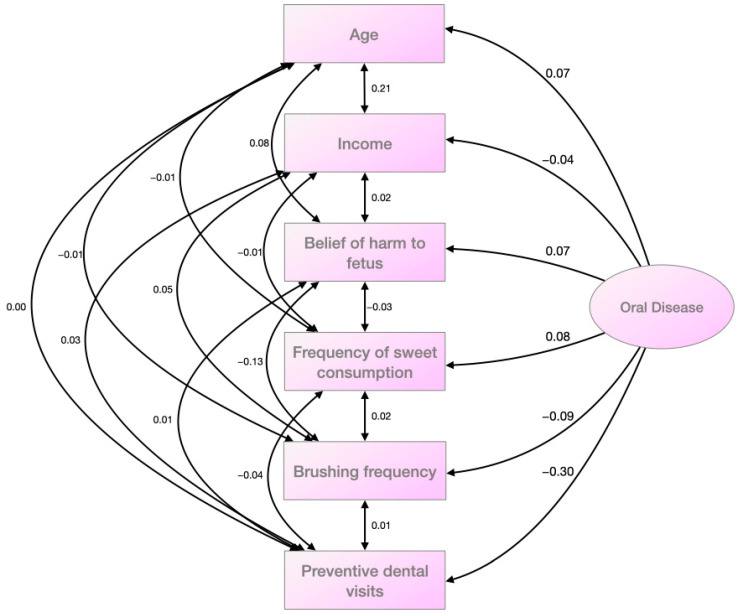
Model obtained after causal path analysis using the significant variables.

To explain the oral disease, a causal path analysis model was performed using significant variables. The model had an R^2^ value of 0.126, indicating its explanatory capacity. The fit to the data had a chi-square value of 0.0. Figure 1 displays the model obtained, along with the estimates. These estimates show the direct effects of independent variables on the dependent variable via regression coefficients. They also indicate the indirect effects of independent variables on each other via covariances. All estimate values are standardized.

The data analyzed showed that the rate of preventive visits and frequent brushing were the two variables that most impacted the oral disease index. The consumption of sweets, age, and the belief that visiting the dentist harms the fetus were also important factors. However, family income had a low explanatory capacity and was almost statistically insignificant. There were no significant covariances when examining the relationships between explanatory variables, except for a few weak ones. Age was associated with income (higher age, higher income), frequency of brushing with fetal harm (higher belief in harm, lower frequency of brushing), and age with fetal harm due to dental visits (higher age, higher belief of harm). Most of the other associations were weak and insignificant.

## 4. Discussion

This research aimed to develop a model explaining the social factors and healthy habits that impact the oral health of expectant mothers in Cuenca, Ecuador. The oral health of the women studied was found inadequate during pregnancy, as well as during the last two years and over their lifetime, according to the frequency of dental visits recorded. Most dental visits (67%) were made during pregnancy due to a mandatory dental check-up program. Despite this program, participants reported a high incidence of oral health problems, contrasting with the low number of visits to the dentist. This finding is particularly concerning given the young age of the sample population.

Considering several significant variables, a causal path analysis model was used to explain the oral disease index. Despite initially including all the correlated variables, we excluded the rinsing frequency variable in the solution presented in the study. Although it correlated significantly with the dependent variable in the exploratory analysis, its regression coefficient was not significant in the model. Moreover, the model improved when we removed it. Similarly, the fear of pain variable did not contribute significantly to the model’s explanatory capacity, although this bivariate correlation was significant. Therefore, we did not include it in the final solution. In the model presented, all regression coefficients were statistically significant.

Our findings suggest that frequent teeth brushing is associated with better oral health. On the contrary, consuming more sweets, being older, and having a higher degree of agreement that visiting the dentist during pregnancy is harmful are associated with an increased risk of oral disease. Surprisingly, family income showed a low impact on oral health. The lack of correlation between educational level and oral health was even more unexpected. These findings are striking as these two factors often contribute to social inequalities in health studies [31,37,38], in oral health [3,9,10,39], or in the oral health of pregnant women [37,40,41]. It is common to find a relationship between socioeconomic position (income, social class and occupation) and health [38,42,43], or with oral health [39,44,45,46]. However, our investigation did not find a significant correlation in our sample. As this model was established using this population as a reference, there are currently no set thresholds. Future studies conducted on other populations may enable the establishment of thresholds when comparing their results to ours. The influence of education, income, and age on oral health can be minimal due to poor preventive practices that affect all social groups. It is common for people only to visit the dentist when they experience pain or have noticeable oral problems. This behavior is widespread in all socioeconomic and educational backgrounds and ages. Visiting a private dentist requires significant financial efforts for families. Although social security may cover dental visits for some social groups, preventive dental care is typically not covered [47]. There is an economic issue facing citizens and a widespread cultural problem in Ecuadorian society due to the lack of regular dental check-ups. Similar studies performed in different countries [48,49] have stated that socioeconomic and educational factors improve the oral health of pregnant women. However, according to our findings, the financial and cultural conditions may affect participants and thus negate the effects of educational status. Therefore, using our model in countries with higher overall socioeconomic status and countries with more robust dental screening plans would be interesting to compare the results.

Our results show that brushing frequency is quite common among the population, ranking as the second most significant factor in maintaining good oral health. However, relying solely on brushing is inadequate in preventing oral problems. In addition, consuming sweets is the third factor in the explanatory model and is a common habit in Ecuadorian society, which counteracts the positive effects of brushing. Pregnant women are often concerned about the potential harm to their fetuses during dental check-ups, particularly during X-rays. This fear is as crucial as age and often leads to postponing dental visits until after childbirth, which can worsen oral health during pregnancy.

Fear of dental pain does not explain oral health status. Although this fear might prevent or postpone dentist visits, the data we collected did not strongly support this hypothesis [50]. Although a variable may be related to the dependent variable when assessing a model, its impact on the overall model can be minimal. Even if the relationship between the two variables is significant, the causal effect is low, which supports the initial hypothesis. We also expected a more significant impact of age, but it should be noted that the sample population in this study is limited to a relatively youthful age range.

The correlations between significant variables were generally low in the model obtained via causal path analysis. This finding may suggest that other variables and factors could be considered in future research. However, we found that the model fit was optimal with the data, as evidenced by a chi-square value of 0.0.

This study has some limitations. First, it is important to acknowledge the presence of a potential selection bias in the study. It is due to the recruitment method that hinders the complete randomization of the sample. While the large number of participants and the detailed description of their demographic characteristics help to evaluate this bias, it is essential to consider this limitation when evaluating the external validity of the study’s conclusions. In this sense, the heterogeneity of the interviewers could have also introduced some potential bias. Another factor to consider is that the participants’ responses may be influenced by bias. They could have overestimated the number of times they brush their teeth and overall oral hygiene or underreported their consumption of sugary foods, as they may not want to reveal bad habits to a health interviewer. Therefore, the findings may only give a partial picture of the oral health issues of the studied population. Finally, it is important to note that the factorial analyses performed have a high sample dependence. Although our sample is large and above the recommendations of other authors [35,51], this aspect should be considered. Another limitation is that we did not analyze the employment status of the participants. Nevertheless, the net monthly income analysis can help overcome this potential limitation. Finally, it is essential to consider that this research has other potential limitations, such as non-response and confounding biases. In this sense, we also should take into account the social desirability bias. Since data collection was conducted face-to-face, regardless of patient anonymity, it is likely that patients were more inclined to give answers that conformed more closely to social expectations. Additionally, the model is robust, and causal relationships cannot be established due to the study’s design.

Our study also has some strengths. The main one is its large sample size, which increases the validity of the findings and reduces the risk of selection bias. In addition, the surveys were administered by trained medical students, ensuring that the data was collected neutrally and objectively. Medical students could also assist participants with questions or concerns, ensuring a clearer understanding of the questions and more accurate responses. Finally, the study gathered responses from real women who identified a significant problem that causes a considerable health gap among women. These findings allow health practitioners, administrators, and policymakers to create interventions at various levels of healthcare care management, potentially improving these women’s lives in their typical environment.

## 5. Conclusions

The most critical determinants of oral health of pregnant women in the canton of Cuenca, Ecuador, are those related to preventive aspects: preventive dentist visits, frequent brushing, and a contained consumption of sweets. The main factors that lead to poorer oral health in pregnant women are their age and the misconception that dental visits can harm their unborn child. Interestingly, income level has a minor impact, while education level and ethnic background seem to have little to no effect. It may be because the lack of regular dental check-ups is widespread across all social groups. These findings are highly relevant, as they have practical applications in clinical and social settings. They can also assist health professionals, managers, and politicians in making informed decisions to improve the oral health of pregnant women. Additionally, this study can be replicated in other countries and cultures to compare the results.

## Figures and Tables

**Table 1:** Main demographic features.

	Income/Level	n	%
Net monthly income	No income	122	6.2
	Lower than 1000 USD	1444	73.3
	1000–1500 USD	232	11.8
	Over 1500 USD	173	8.8
Education level	Primary studies	616	31.3
	Secondary studies	860	43.6
	University studies	495	25.1

USD: U.S. Dollar.

**Table 2:** Incidence of oral health problems in pregnant women.

Oral Health Problem	P1.a (n)	P1.b (%)	P1.c (n)	P1.d (%)
Toothache	74	33	21	45
Cavities	84	58	34	74
Crooked teeth	38	18	11	16
Bad breath	27	14	8	5
Loss of permanent teeth	28	8	3	13
Having phlegmon, having a swollen face due to a tooth	16	5	2	6
Earache or hinge pain due to a bad bite	18	7	3	4
Bleeding gums from brushing or biting on food	47	33	33	16
Gingivitis (gum inflammation due to dental plaque or tartar)	26	14	14	12
Pyorrhea or periodontitis (gum recedes, leaving loose teeth)	5	3	2	2
They do not mention any problems	1	3	22	4

P1.a: Which of the following oral health problems have you had at least once in your life?; P1.b: Of these problems, what have you had in the last two years?; P1.c: Of these problems, which ones have you had during your pregnancy?; P1.d: Of these problems, what have you ever had when visiting a dentist?

**Table 3:** Components matrix *.

Title 1	Title 3
P1.a Gingivitis	0.560
P1.a Pyorrhea or periodontitis	0.553
P1.b Pyorrhea or periodontitis	0.547
P1.b Gingivitis	0.520
P1.c Gingivitis	0.497
P1.c Pyorrhea or periodontitis P1.c Periodontitis or pyorrhea	0.491
P1.a Bleeding gums from brushing or biting on food P1.b Bleeding gums from brushing or biting on food P1.c Gingivitis	0.455
P1.a Bad breath	0.452
P1.b Bad breath	0.433
P1.a Have a boil, have a swollen face from a tooth P1.b Bad breath	0.424
P1.a Teeth that are loose or crooked	0.413
P1.b Bleeding gums from brushing or biting on food P1.b Teeth that are loose or crooked	0.398
P1.b Teeth loose, crooked, or buckled teeth	0.379
P1.c Bleeding gums from brushing or biting food P1.c Teeth loose or crooked	0.352
P1.c Teeth loose or crooked	0.330
P1.a Loss of permanent teeth P1.b Loss of permanent teeth	0.323
P1.a Ear or hinge pain due to bad bite P1.c Bad breath	0.303
P1.c Bad breath	0.291
P1.b Loss of permanent teeth P1.c Bad breath	0.252
P1.b Have a boil, have a swollen face due to a tooth P1.b Caries	0.211
P1.b Cavities	0.208
P1.b Ear or hinge pain due to a bad bite P1.a Ear or hinge pain due to bad bite	0.191
P1.a Toothache	0.159
P1.c Phlegmon, swelling of the face due to a tooth P1.b Caries	0.133
P1.a Tooth decay	0.132
P1.b Toothache	0.110

* Cronbach Alpha: 0.721. KMO = 0.691. Barlett’s sphericity test: Chi-square *p* = 0.0000. Self-value: 3.701 (14.23%). Extraction: principal components by forcing 1 factor.

**Table 4:** Items selected for the index.

Item	P1.a	P1.b	P1.c
Toothache	X	X	
Cavities	X	X	
Crooked teeth	X	X	X
Bad breath	X	X	X
Loss of permanent teeth	X	X	
Having phlegmon, having a swollen face due to a tooth	X	X	X
Earache or hinge pain due to a bad bite	X	X	
Bleeding gums from brushing or biting on food	X	X	X
Gingivitis (gum inflammation due to dental plaque or tartar)	X	X	X
Pyorrhea or periodontitis (gum recedes, leaving the teeth loose)	X	X	X

P1.a: Which of the following oral health problems have you had at least once in your life?; P1.b: Of these problems, tell me which ones you have had in the last two years.); P1.c: Of these problems, tell me which ones you have had during your current pregnancy.

**Table 5:** Regression weights.

		Estimate	S.E. *	C.R. *	*p*-Value	Label
Oral Disease	Harm Fetus	0.081	0.023	3.493	***	par_6
Oral Disease	Preventive visits	−1.689	0.118	−14.301	***	par_7
Oral Disease	Age	0.012	0.003	3.382	***	par_17
Oral Disease	Income	−0.118	0.058	−2.023	0.043	par_18
Oral Disease	Brushing frequency	−0.098	0.022	−4.430	***	par_19
Oral Disease	Sweet consumption	0.278	0.071	3.919	***	par_21

* SE = Standard errors. C.R. = Critical Ratios. *** *p* < 0.001.

**Table 6:** Standardized regression weights.

		Estimate
Oral Disease	Harm Fetus	0.075
Oral Disease	Preventive visits	−0.302
Oral Disease	Age	0.073
Oral Disease	Income	−0.044
Oral Disease	Brushing frequency	−0.094
Oral Disease	Sweet consumption	0.083

**Table 7:** Covariances.

		Estimate	S.E. *	C.R. *	*p*-Value	Label
Brushing frequency	Preventive visits	0.002	0.004	0.499	0.618	par_1
Brushing frequency	Harm Fetus	−0.111	0.020	−5.555	***	par_2
Brushing frequency	Income	0.018	0.008	2.297	0.022	par_3
Harm Fetus	Age	0.453	0.130	3.479	***	par_4
Income	Age	0.497	0.054	9.265	***	par_5
HARMFETUS	Income	0.006	0.008	0.825	0.409	par_8
Preventive visits	Harm Fetus	0.002	0.004	0.660	0.509	par_9
Preventive visits	Income	0.002	0.001	1.341	0.180	par_10
Preventive visits	Age	0.002	0.025	0.066	0.948	par_11
Brushing frequency	Age	−0.030	0.136	−0.223	0.824	par_12
Income	Sweet consumption	−0.001	0.002	−0.485	0.628	par_13
Age	Sweet consumption	−0.010	0.042	−0.233	0.816	par_14
Preventive visits	Sweet consumption	−0.002	0.001	−1.713	0.087	par_15
Brushing frequency	Sweet consumption	0.006	0.006	0.879	0.379	par_16
Harm Fetus	Sweet consumption	−0.007	0.006	−1.138	0.255	par_20

* SE = Standard errors. C.R. = Critical Ratios. *** *p* < 0.001.

**Table 8:** Squared multiple correlations.

		Sweet Consumption	Age	Income	Harm Fetus	Preventive Visits	Brushing Frequency
Oral disease	Total Effects	0.278	0.012	−0.118	0.081	−1.689	−0.098
	Standardized Total Effects	0.083	0.073	−0.044	0.075	−0.302	−0.094
	Direct Effects	0.278	0.012	−0.118	0.081	−1.689	−0.098
	Standardized Direct Effects	0.083	0.073	−0.044	0.075	−0.302	−0.094
	Indirect Effects	0.000	0.000	0.000	0.000	0.000	0.000
	Standardized Indirect Effects	0.000	0.000	0.000	0.000	0.000	0.000

Estimate 0.124.

**Table 9:** Pearson’s correlations between oral diseases and independent variables.

Dependent Variable	Statistic	Value
Indigenous	Pearson’s correlation	0.043
	Sig. (bilateral)	0.059
	N	1971
Age	Pearson’s correlation	0.068 **
	Sig. (bilateral)	0.003
	N	1971
Income	Pearson’s correlation	−0.049 *
	Sig. (bilateral)	0.029
	N	1971
Education level	Pearson’s correlation	−0.031
	Sig. (bilateral)	0.165
	N	1971
Brushing frequency	Pearson’s correlation	−0.098 **
	Sig. (bilateral)	0
	N	1971
Rinsing frequency	Pearson’s correlation	−0.061 **
	Sig. (bilateral)	0.007
	N	1971
Frequency of sweet consumption	Pearson’s correlation	0.075 **
	Sig. (bilateral)	0.001
	N	1971
The belief of harm to the fetus	Pearson’s correlation	0.086 **
	Sig. (bilateral)	0
	N	1971
Fear or pain	Pearson’s correlation	0.074 **
	Sig. (bilateral)	0.001
	N	1971
Preventive dental visits	Pearson’s correlation	−0.303 **
	Sig. (bilateral)	0
	N	1971

** Correlation is significant at the 0.01 level (bilateral). * Correlation is significant at the 0.05 level (bilateral).

## Data Availability

The data presented in this study are available on request from the corresponding author.
